# CpG site methylation in *CRYAA* promoter affect transcription factor Sp1 binding in human lens epithelial cells

**DOI:** 10.1186/s12886-016-0309-y

**Published:** 2016-08-09

**Authors:** Xin Liu, Peng Zhou, Fan Fan, Dan Li, Jihong Wu, Yi Lu, Yi Luo

**Affiliations:** 1 Department of Ophthalmology, Eye and ENT Hospital of Fudan University, 83 FenYang Road, Shanghai, 200031 People’s Republic of China; 2 Key Laboratory of Myopia, Ministry of Health, 83 FenYang Road, Shanghai, 200031 People’s Republic of China; 3 Shanghai Key Laboratory of Visual Impairment and Restoration, Fudan University, 83 FenYang Road, Shanghai, 200031 People’s Republic of China; 4 Department of Ophthalmology, Parkway Health, Specialty and Inpatient Center (Luwan), 170 DanShui Road, Floor 3, Shanghai, 200020 People’s Republic of China; 5 Hong Qiao Medical Center, 2258 HongQiao Road, Shanghai, 200033 People’s Republic of China

**Keywords:** DNA methylation, CRYAA, Transcription factor Sp1, Human lens epithelial cells, Zebularine

## Abstract

**Background:**

Age-related cataract (ARC) is the leading cause of visual impairment worldwide, and α-crystallin (*CRYAA*) is the predominant structural protein involved in the maintenance of lens clarity and refractive properties. We previously demonstrated that *CRYAA* genes undergo epigenetic repression in the lens epithelia in ARC. We further analyze the underlying mechanism in the current study.

**Methods:**

The transcription factor binding sites of the CpG island of *CRYAA* promoter were predicted by TESS website. An electrophoretic mobility shift assay (EMSA) was used to analyze the impact of the methylation of CpG sites on transcription factors. Human lens epithelial B-3 (HLE B-3) Cells were treated with demethylation agent zebularine in the concentrations of 0 (PBS as control), 10 μM, 20 μM, 50 μM, 100 μM and 200 μM, respectively. After treatment in the above concentrations for 24 h, 48 h and 72 h, respectively, *CRYAA* mRNA expression levels were detected by Quantitative Real-Time RT-PCR.

**Results:**

The methylation of the CpG site of the *CRYAA* promoter decreased the DNA-binding capacity of transcription factor Sp1. Zebularine increased *CRYAA* expression in HLE B-3 Cells in a dose- dependent and time- dependent pattern.

**Conclusions:**

The evidence presented suggests that the methylation of the CpG sites of the *CRYAA* promotor directly affect Sp1 binding, leading to down expression of *CRYAA* in human lens epithelial cells. Zebularine treatment could restore *CRYAA* expression in a dose- dependent and time- dependent pattern.

**Electronic supplementary material:**

The online version of this article (doi:10.1186/s12886-016-0309-y) contains supplementary material, which is available to authorized users.

## Background

Age-related cataract (ARC) is the leading cause of visual impairment among older adults [1, 2]. It is widely accepted that senescence, heredity, and environment are the major contributing factors in ARC and that oxidative stress plays an important role in cataractogenesis [3, 4]. However, the precise mechanism of ARC remains to be further elucidated.

Lens crystallins are the predominant structural proteins involved in the maintenance of lens clarity and refractive properties [5, 6]. α-crystallins constitute 35 % of all crystallins [7]. Its molecular chaperone-like activity protects other crystallins against thermal-induced inactivation or aggregation and allows the lens to resist the aging-induced deterioration of proteins [8]; therefore it is thought to be critical for maintaining lens transparency. Our previous studies have shown, for the first time, that DNA methylation regulates gene expression in lens epithelial cells [9, 10]. *CRYAA* undergoes epigenetic repression in the lens epithelia in nuclear ARC [11]. However, the mechanism of *CRYAA* DNA methylation remains to be explored.

Epigenetic modifications are post-transcriptional, reversible, and hereditable events that do not alter the genetic sequence. The epigenetic regulation of gene expression mainly includes DNA methylation, histone modification, and non-coding RNA [12]. DNA methylation, mostly in the form of 5′-methylcytosine in CpG dinucleotides in the presence of DNA methyltransferases (DNMTs), often acts as a transcriptional repressor [13]. It can directly prevent the binding of transcription factors or act through interactions with methyl-CpG-binding domain (MBD) proteins and histone deacetylases (HDACs), resulting in gene silencing [14].

In the present study, we analyzed the impact of the methylation of CpG sites on transcription factors to explore the underlying mechanism of the down regulation of *CRYAA* in nuclear ARC. We also investigated the effect of DNA-demethylating agent Zebularine on the expression of *CRYAA*.

## Methods

All procedures were performed in accordance with the tenets of the Declaration of Helsinki. The institutional review board of the Eye and ENT Hospital of Fudan University approved our use of cultured human lens epithelial cells.

### Human lens epithelium B-3 (HLE B-3) cell culture

HLE B-3 cells were obtained from American Type Culture Collection (ATCC; Rockville, MD, USA) and cultured in Eagle’s minimum essential medium (Gibco-BRL, Grand Island, NY, USA) with 20 % FBS, 100 U/ml penicillin, and 100 mg/ml streptomycin at 37 °C in a humidified 5 % CO_2_ atmosphere.

### In silico analysis

The transcription factor binding sites of the CpG island of *CRYAA* promoter were predicted by TESS website (http://www.cbil.upenn.edu/cgi-bin/tess/tess) as described before [15].

### Electrophoretic Mobility Shift Assay (EMSA)

EMSA was conducted as described previously, with minor changes [15, 16]. Nuclear proteins were extracted from HLE B3 cells using NE-PER nuclear and cytoplasmic extraction reagents (Pierce Biotechnology, Rockford, IL, USA). Methylated and wild-type oligonucleotides, were purchased from Shenggong Technologies, Inc. (China). These included the wild-type SP-1 consensus binding sequence of the CRYAA promoter (5′ –GGCTGGGCGTCCA-3′) and the methylated Sp1 consensus binding sequence (5′ –GGCTGGGC^m^GTCCA-3′). The Sp1 consensus binding site is underlined. All listed oligonucleotides included antisense oligonucleotide strands. In methylated oligonucleotides, the antisense oligonucleotide strands were methylated at the cytosine corresponding to that of the sense strand. EMSA probes were synthesized as double strands after pair annealing and 3′-end-labeled with biotin (Invitrogen). Unlabeled oligonucleotides with identical sequences were used as competitors. The EMSA was performed using the LightShift Chemiluminescent EMSA kit (Pierce Biotechnology), following the manufacturer’s protocol. For supershift experiments, Sp1 antibody (Abcam) was added to the reaction solution 30 min prior to the addition of the probes. The densities (total gray) of the bands were calculated using Glyko Bandscan 5.0 software (ProZyme, Hayward, CA, USA).

### Zebularine treatment

A demethylating agent, 1-(β-D-ribofuranosyl)-1,2-dihydropyrimidin-2-one, or zebularine (Sigma), was used to investigate its effect on the expression of HLE B-3 cells as described previously [17]. Cells were plated at a density of 2*10^5^ per well in six-well plates 24 h before treatment. Cells were treated with 0 (PBS as control), 10 μM, 20 μM, 50 μM, 100 μM and 200 μM Zebularine, respectively. After treatment in the above concentrations for 24 h, 48 h and 72 h, respectively, the cells were harvested.

### Quantitative real-time RT-PCR

Total RNA was isolated from HLE B-3 cells after Zebularine treatment using RNeasy Mini Kit (Qiagen, Valencia, CA, USA) according to the manufacturer’s instructions. A total of 1 μg total RNA extracted was reverse transcribed with the PrimeScript™ RT Master Mix (Takara Bio, Inc., Shiga, Japan) in accordance with the manufacturer’s protocol. [18] CRYAA mRNA expression were detected using a SYBR Green detection kit (SYBR Premix Ex Taq; Takara, Osaka, Japan) as described previously [19]. β-actin was used as an internal control. The primers used in qRT-PCR were as follows: (a) Gene “Homo sapiens crystallin alpha A (CRYAA), mRNA”, the NCBI Reference Sequence is NM_000394.3“; Forward primer “GAAGGTGCAGGACGACTTTG” is located from 273 nt to 292 nt of the NM_000394.3 sequence; Reverse primer “CAGAAGGTCAGCATGCCATC” is located from 437 nt to 428 nt of the NM_000394.3 sequence. (b) Gene “Homo sapiens ACTB actin, beta, mRNA”, the NCBI Reference Sequence is NM_001101.3”; Forward primer “ CAACTCCATCATGAAGTGTGACG” is located from 921 nt to 943 nt of the NM_001101.3 sequence; Reverse primer “ ACTCGTCATACTCCTGCTTGC” is located from 1177 nt to 1157 nt of the NM_001101.3 sequence. qRT-PCR reactions were performed with a ViiA 7 Real-Time PCR System (Life Technologies, Pleasanton, CA). Relative fold changes in CRYAA mRNA expression were determined by calculating 2^-ΔΔCT^ with ViiA 7 Software (Life Technologies). All qRT-PCR experiments were performed for three times, with biological triplicates in each experiment.

### Statistical analysis

The results of the experiments are expressed as means ± SD. A student’s *t*-test or the Mann–Whitney test was used to compare quantitative data between two groups. A value of *P* < 0.05 was considered significant.

## Results

### Transcription factor binding sites prediction

**Fig. 1:**
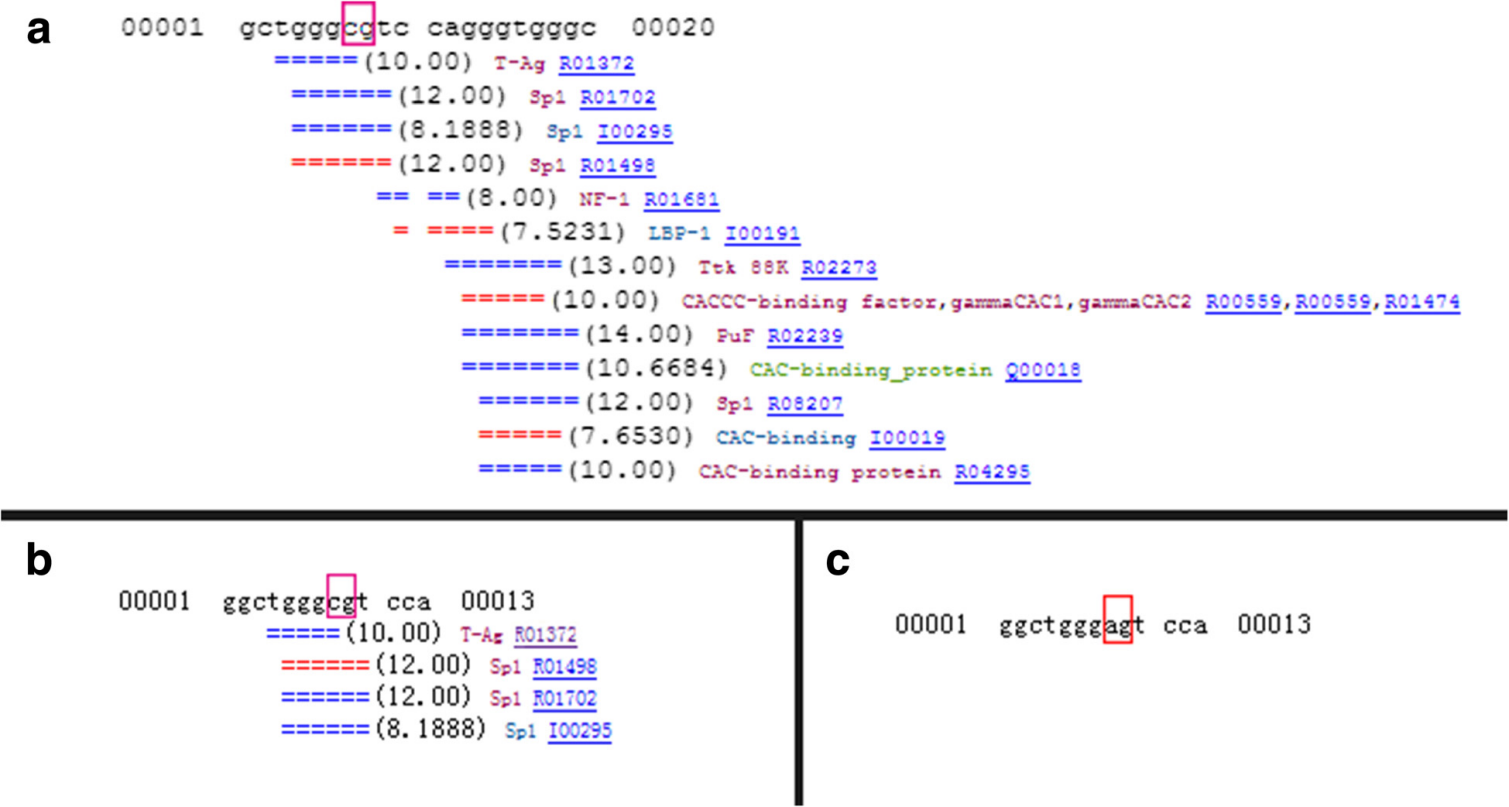
Transcription factor binding sites Prediction. The sequence around the CpG site, which displays the most significant differences in methylation between nuclear ARC cases and controls, is analyzed for transcription factor binding prediction via TESS website. **a** Several transcription factor-binding sites are predicted in the sequence. The most frequent predicted transcription factor is Sp1. **b** Transcription factor binding in seleted sequence. We shorten the sequence and select one, which mainly binds to Sp1 for further study. **c** Transcription factor binding in mutated sequence. If the “cg” site mutate to “ag” site, no transcription factor binding is found

Our previous study demonstrated that CpG sites in the CpG island of CRYAA promoter were hypermethylated in the lens epithelia of nuclear ARC cases versus age- matched controls [11]. The sequence around the CpG site, which displayed the most significant differences in methylation between nuclear ARC cases and controls, was analyzed for transcription factor binding prediction via TESS website. Several transcription factor-binding sites were predicted in the sequence (Fig. 1a). The most frequent predicted transcription factor was Sp1. A selected sequence, which mainly bound to Sp1, was chosen for further study (Fig. 1b). If this sequence was mutated, no transcription factor binding was found (Fig. 1c). As the effect of methylation on transcription factor could not be predicted by the website, we used the model of mutated sequence to predict the effect of change of the CpG site to the binding of transcription factor. According to the prediction results, we hypothesized that transcription factor binding would be affected after methylation of the CpG site. We used EMSA to further confirm our prediction and hypothesis.

### CpG methylation of *CRYAA* promoter decreases the DNA-binding capacity of transcription factor Sp1

**Fig. 2:**
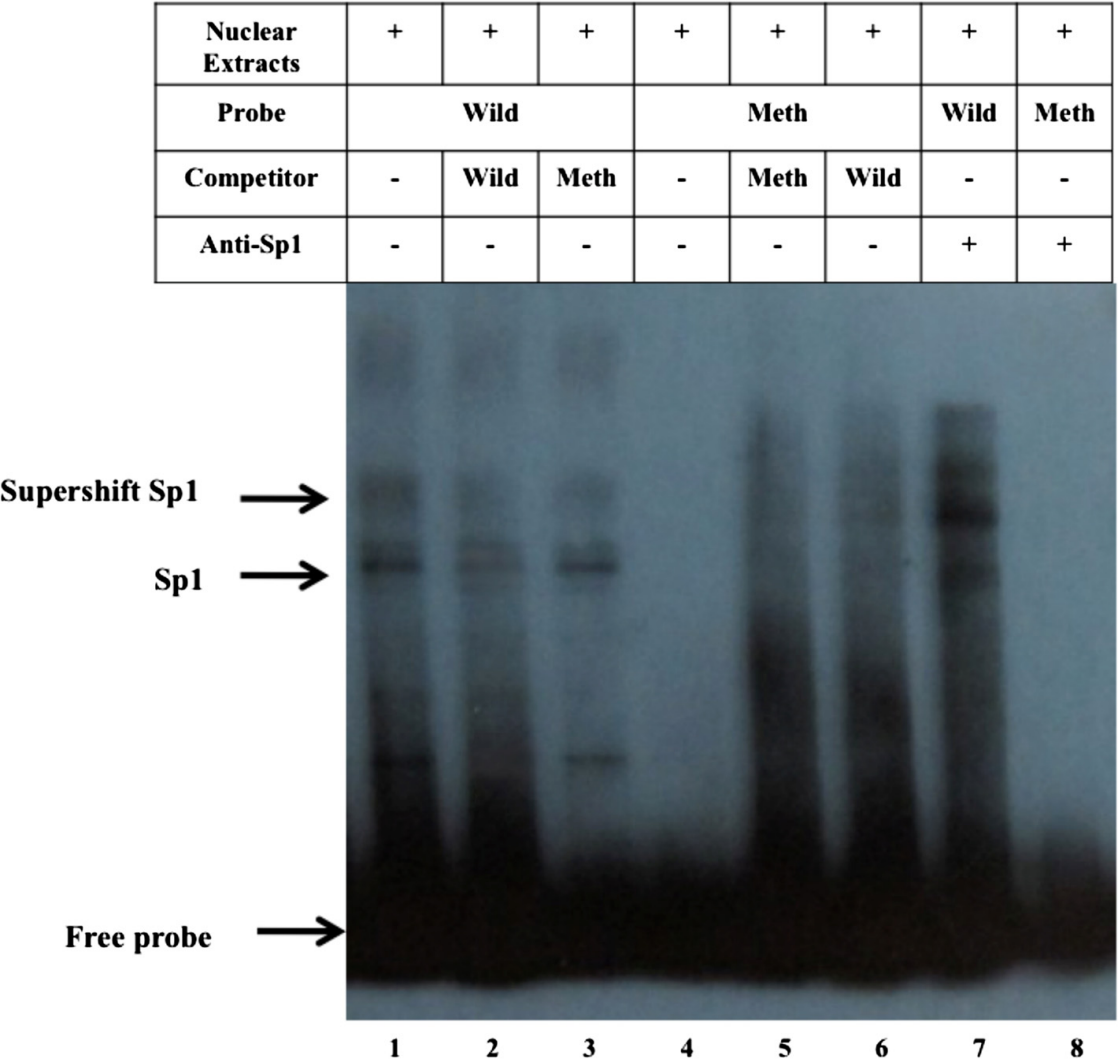
CpG methylation of CRYAA promoter decreases the DNA-binding capacity of transcription factor SP-1. EMSA experiments show the specific binding of Sp1 to the binding sequence of the CRYAA promoter. Lane 1: biotin-labeled wild-type Sp1 probe incubated with HLE B-3 nuclear extracts. Sp1 binding is depicted by arrows on the left of the figures. Lane 2: excess unlabeled wild-type competitor (1:100) competes for binding with the labeled probe. Lane 3: unlabeled methylated competitor (1:100) does not compete with the labeled probe. Lane 4: labeled methylated probe incubates with nuclear extracts. No binding was shown. Lanes 5 and 6: excess unlabeled wild-type and methylated competitors (1:100) were added, respectively. No binding of Sp1 was shown. Lane 7 shows anti-Sp1 supershift. Anti-Sp1 antibody is added to nuclear extracts incubating with a wild-type probe. Lane 8 shows no binding of Sp1. Anti-Sp1 antibody is added to nuclear extracts incubating with a methylated probe

EMSA was performed to determine whether the methylation of the CpG sites of the *CRYAA* promoter influences the binding of transcription factor Sp1 to its consensus binding sequence. A labeled wild-type probe containing the Sp1 consensus binding sequence (GGGCGT) of the *CRYAA* promoter was used to test Sp1 binding. The incubation of HLE B-3 nuclear extracts with a wild-type Sp1 probe revealed a pattern of shifted bands representing Sp1 binding activity. HLE B-3 nuclear extracts were able to bind the wild-type Sp1 probe (lane 1), while the binding capacity significantly decreased when incubated with a methylated probe (lane 4 and 8). The unlabeled wild-type Sp1 probe can sufficiently compete with the labeled wild-type probe (lane 2), while the unlabeled methylated probe could not compete (lane 3). When Sp1 antibody was added to the incubation mixture, Sp1-specific binding was demonstrated via the decreased intensity of the Sp1 bands, as well as by a supershift seen in lane 7 (Fig. 2). This result suggests that the hypermethylation of the CpG sites of the *CRYAA* promoter decreases the DNA-binding capacity of transcription factor Sp1.

### Demethylation using zebularine increased *CRYAA* expression in a dose- dependent and time- dependent pattern in HLE B-3 cells

**Fig. 3:**
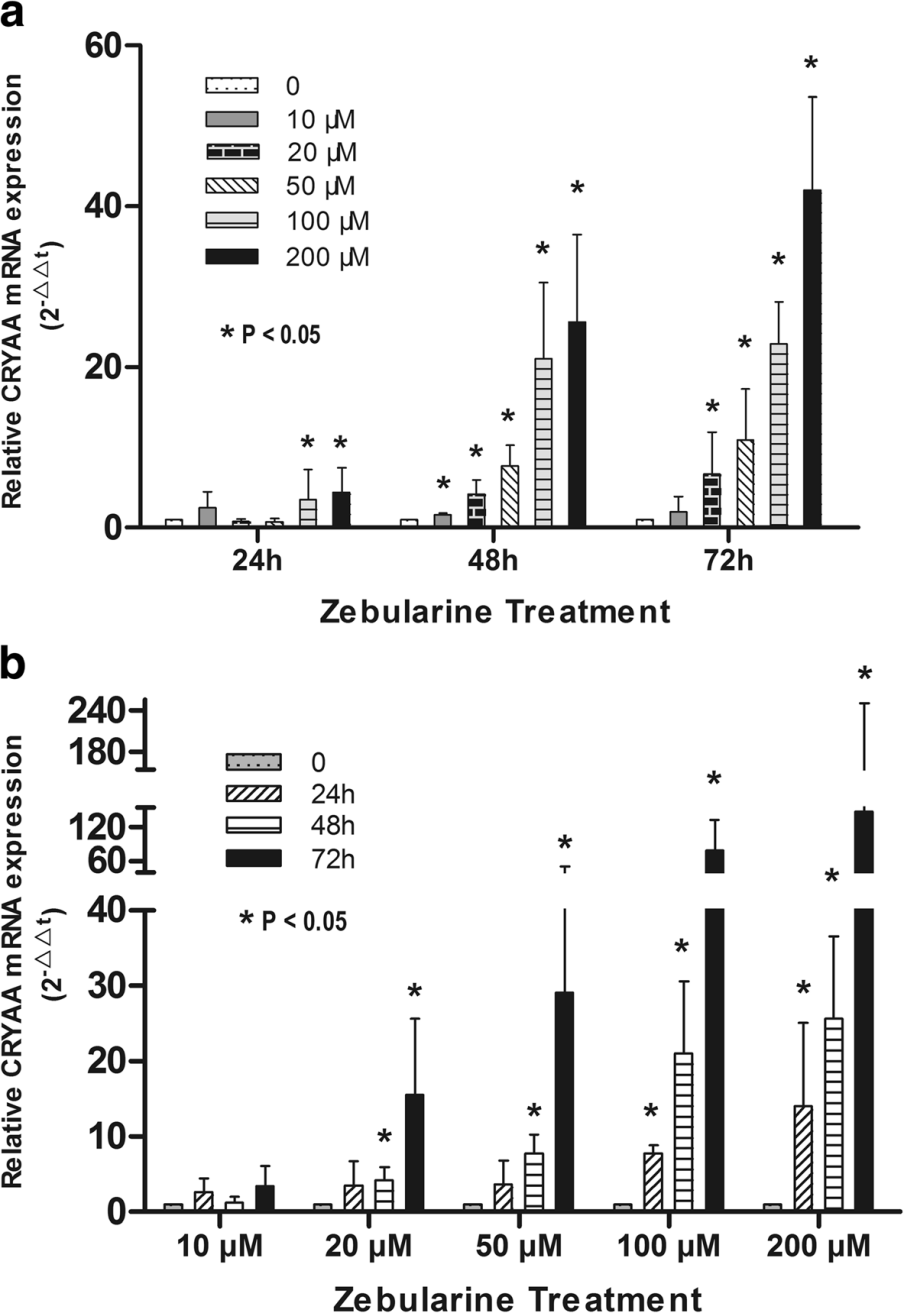
Demethylation agent Zebularine increased CRYAA mRNA expression in HLE B-3 cells detected by Real time qRT-PCR. A. Zebularine increased CRYAA mRNA expression in a dose-dependent pattern. As the concentration of zebularine increased from 10 μM to 200 μM, the CRYAA mRNA level increased to 4.5 fold of untreated controls after zebularine treatment for 24 h, 25.6 fold for 48 h and 42.0 fold for 72 h. B. Zebularine also increased CRYAA mRNA expression in a time-dependent pattern. After 24 to 72 h with zebularine treatment of different concentrations, the CRYAA mRNA expression level increased to 3.4 fold of the control level in 10 μM concentration, 15.6 fold in 20 μM, 29.1 fold in 50 μM, 78.8 fold in 100 μM and 147.3 fold in 200 μM. *P < 0.05. The error bars represent standard deviation of the mean of 3 experiments

We analyzed treatment of demethylation agent zebularine on CRYAA mRNA expression in lens epithelial cells. Our results indicated that zebularine increased CRYAA mRNA expression in a dose-dependent pattern. As the concentration of zebularine increased from 10 μM to 200 μM, the CRYAA mRNA level increased to 4.5 fold of untreated controls after zebularine treatment for 24 h, 25.6 fold for 48 h and 42.0 fold for 72 h (*P* < 0.05) (Fig. 3a). Zebularine also increased CRYAA mRNA expression in a time-dependent pattern. After 24 to 72 h with zebularine treatment of different concentrations, the CRYAA mRNA expression level increased to 3.4 fold of the control level in 10 μM concentration, 15.6 fold in 20 μM, 29.1 fold in 50 μM, 78.8 fold in 100 μM and 147.3 fold in 200 μM (*P* < 0.05) (Fig. 3b). These results suggested that demethylation could lead to upregulation of *CRYAA* expression in a dose- dependent and time- dependent pattern (Additional files 1, 2, 3 and 4).

## Discussion

Diverse DNA methylation patterns have recently been discovered in many ocular diseases, including glaucoma [20], age-related macular degeneration (AMD) [21], retinoblastoma [22–25], uveal melanoma [26–29], and cataract [11, 30]. Our previous work demonstrated, for the first time, that DNA methylation down-regulates *CRYAA* gene expression in lens epithelial cells in nuclear ARC [9–11]. However, little is known about the precise mechanism of DNA methylation of *CRYAA*. In this study, we showed that in lens epithelial cells, the methylation of the CpG site of the *CRYAA* promoter decreased the DNA-binding capacity of transcription factor Sp1. Treatment with the DNA-demethylating agent Zebularine increased *CRYAA* expression in a dose- dependent and time- dependent pattern. Overall, these findings suggest that the methylation of the CpG sites of the *CRYAA* promotor directly affects transcription factor binding and is related to the epigenetic repression in nuclear ARC lenses. We have demonstrated the underlying mechanism of the DNA methylation of *CRYAA* in HLE B-3 cells.

DNA methylation may be an important mechanism in the pathogenesis and progression of cataract. Critical enzymes involved in DNA methylation, such as DNMT1 and MeCP2, were found in human lens epithelial cells [9, 10]. The down-regulation of *CRYAA* via the hypermethylation of CpG islands in its promoter was found in nuclear ARC cases [11]. It was also reported to be related to the earlier onset of dark nucleus in highly myopic patients [31], as well as nuclear cataract formation after pars plana vitrectomy [32]. It is established that the localized reduction of antioxidative capacity in the nuclear region of the lens results in increasing numbers of denatured proteins [3]. Down-regulation via the promoter hypermethylation of *CRYAA* reduces the expression of chaperones, which are able to bind to these denatured proteins and thus preserve the transparency of the lens [6]. This may accelerate the oxidative modification of proteins in the nucleus, resulting in the pathogenesis of nuclear cataract [31]. The dysfunction of Nrf2-dependent antioxidant protection via endoplasmic-reticulum-associated degradation and redox- balance alteration in the lens due to the demethylation of the CpG islands in the *Keap1* promoter is linked to diabetic cataracts and ARCs in both human lens epithelial cells and animal models [30, 33, 34]. The DNA hypermethylation of the promoter region of the DNA repair gene *MGMT* may regulate the down-expression of the gene and be involved in the development of ARC [35]. In cortical ARC, the loss of functional *OGG1* via the base excision repair pathway results in oxidative DNA base damage [36, 37]. Reduced *OGG1* expression was correlated with the hypermethylation of a CpG island of *OGG1* in ARC lenses [38]. Pseudoexfoliation syndrome (PEX)-complicated cataracts also underwent epigenetic regulation. The susceptible PEX gene LOXL1 was hypermethylated in its promoter region and was down- regulated on the mRNA and protein level in Uighur PEX cataract patients [39]. Together, these results suggest that many critical genes related to antioxidative capacity and DNA repair underwent epigenetic repression during the pathogenesis of ARC. In this study, we demonstrated that demethylation treatment with Zebularine increased *CRYAA* expression level in a dose- dependent and time- dependent pattern.

The methylation status of critical CpG sites often conversely correlates with the transcriptional activity of promoters. Two modes exist that explain how the methylation of CpG sites interferes with transcription. Methylated CpG sites can directly interfere with the binding of transcription factors to their recognition sites or facilitate the binding of a family of methyl-binding proteins to their cognate DNA sequences [40]. In this study, we demonstrated that the methylation of the CpG sites of the CRYAA promoter could directly interfere with the binding of transcription factor Sp1 to its recognition elements, which is related to gene repression. Our results were consistent with the previous findings. Clark SJ et al. [41] discovered that ^m^Cp^m^CpG methylation could have a biological function in preventing Sp1 binding, thereby contributing to the subsequent abnormal methylation of CpG islands often observed in tumor cells. Zhu WG et al. [16] demonstrated that hypermethylation around consensus Sp1-binding sites may directly reduce Sp1/Sp3 binding, leading to reduced p21^Cip1^ expression in response to depsipeptide treatment. Zelko et al. [42] found that CpG methylation attenuates Sp1 and Sp3 binding to the human extracellular superoxide dismutase promoter and regulates its cell-specific expression. Douet et al. [43] provided the first direct evidence that CpG methylation of the *Abcc6* proximal promoter region regulates the binding of transcription factor Sp1 and participates in tissue-specific expression control in mice. Li et al. [44] demonstrated that treatment with the demethylation agent 5-aza-2′-deoxycytidine markedly enhanced the binding affinity of Sp1/Sp3 to the promoter region and restored the expression of *CIDE-A* gene in cells. This treatment was also effective in the restoration of the binding of Sp1 to the promoter, as well as *Keap1* expression, in an A549 cell line [45]. In the current study, the excess wild-type competitors did not completely competed away the binding of SP1 in Lane 2. HLE B-3 nuclear extracts were able to bind both the labeled and unlabeled wild-type Sp1 probe. When excess unlabeled wild-type Sp1 probe (100 fold) was used to compete with the labeled wild-type probe, most Sp1 and other transcription factors binded to the unlabeled wild-type Sp1 probe. However, the binding capacity of Sp1 and the wild-type probe was high. The low concentration of labeled wild-type probe could still bind to some SP1. The result indicated that the binding capacity of the low concentration of wild-type probe with SP1 was higher than the normal concentration of the methylated probe. It further confirmed that the methylation of the CpG sites of the *CRYAA* promoter influences the binding capacity of transcription factor Sp1. These results indicate that CpG methylation plays an important role in establishing and maintaining the tissue- and cell-specific transcription of genes through the direct regulation of Sp1 binding.

In this study, we did not investigate the possibility of *de novo* DNA methylation occurring. Further studies should focus on the pathway that affects DNA methylation. Performing bisulphite analysis to analyze the methylation status of the CpG sites around this SP1 binding site will further increase the significance of the study.

## Conclusion

**Fig. 4:**
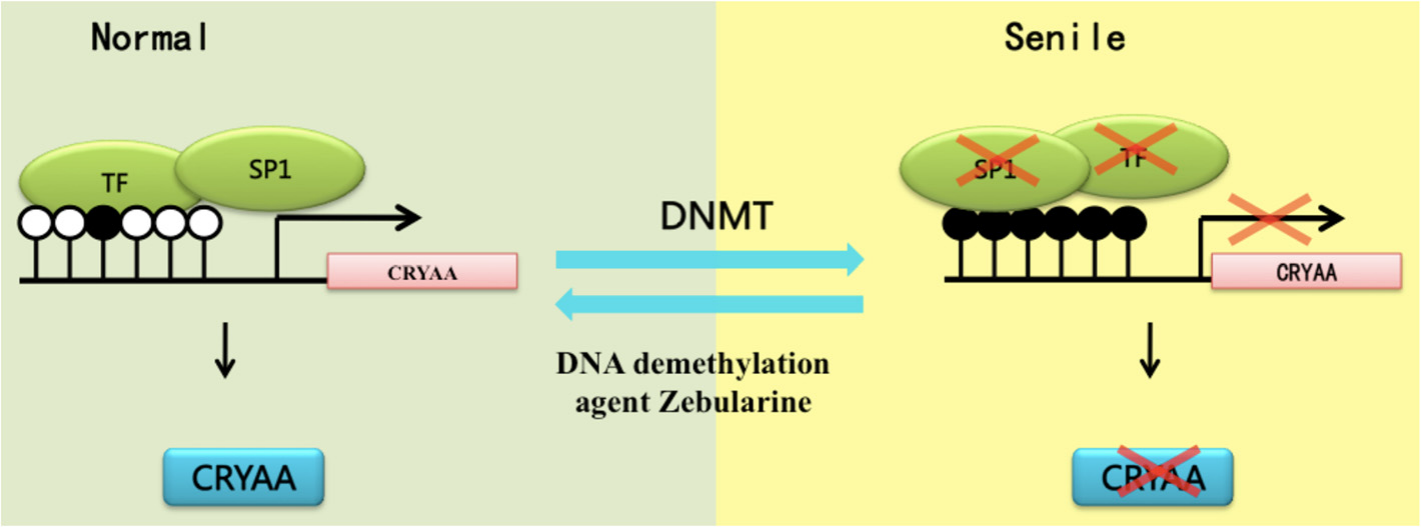
Schematic illustration of the mechanism of DNA methylation of CRYAA in nuclear ARC. Under normal condition, only a few CpG sites are methylated, DNA binds to transcription factors, and CRYAA gene transcription is normal. With aging, the CpG sites of the CRYAA promoter become hypermethylated. Transcription factors, especially Sp1, cannot bind to DNA sequences. Therefore, gene transcription and expression are down-regulated. These procedures are reversible via the DNA demethylation agent Zebularine

The present study demonstrated that the methylation of the CpG site of the CRYAA promoter decreased the DNA-binding capacity of transcription factor Sp1. Treatment with the DNA-demethylating agent Zebularine increased *CRYAA* expression in HLE B-3 Cells in a dose- dependent and time- dependent pattern. Overall, these findings suggest that the methylation of the CpG sites of the CRYAA promotor directly affect Sp1 binding, leading to epigenetic repression in nuclear ARC lenses (Fig. 4). The present study provides some basis for further understanding of the mechanism evolving the epigenetic pathogenesis of nuclear ARC, and it is promising for exploration of novel cataract therapies.

## Additional files

Additional file 1: Row data of qRT-PCR for CRYAA after Zebularine treatment, Experiment 1, Part 1. CRYAA mRNA expression were detected using a SYBR Green detection kit. β-actin was used as an internal control. qRT-PCR reactions were performed with a ViiA 7 Real-Time PCR System. All qRT-PCR experiments were performed for three times, with biological triplicates in each experiment. It is the rata data of part 1 of the first experiment. (XLS 2430 kb) Additional file 2: Row data of qRT-PCR for CRYAA after Zebularine treatment, Experiment 1, Part 2. It is the rata data of part 2 of the first experiment described above. (XLS 2440 kb) Additional file 3: Row data of qRT-PCR for CRYAA after Zebularine treatment, Experiment 2. It is the rata data of the second experiment described above. (XLS 2431 kb) Additional file 4: Row data of qRT-PCR for CRYAA after Zebularine treatment, Experiment 3. It is the rata data of the third experiment described above. (XLS 2630 kb)
